# Antagonism of Typhoid and Intermittent Fevers

**Published:** 1844-03-09

**Authors:** 


### ANTAGONISM OF TYPHOID AND INTERMITTENT FEVERS.

At the séance of the Academie de Médicine, on 21st November, a letter was read from M. Boudin, chief physician to the Military Hospital at Versailles, of which we extract the following summary from the "Gazette Médicale" of Nov. 25:

There is going on at this moment, under the very walls of Paris, a pathological phenomenon, which sheds no small light on one of the most important and deeply-agitated medical questions of the present day.

There are, by chance, at Courbevoie, one league from Paris, in the same barrack, two infantry regiments, which, since their *reunion*, have perpetually presented the most opposite pathological diathesis.

Thus, one of these regiments, the 23d, affords plenty of cases of typhoid fever, and scarcely any of intermittent; whilst the other, the 69th, has for the last seven months kept the hospital at Versailles full of intermittent cases, but has only presented two cases of typhoid fever.

Since the *existing* hygienic conditions of both regiments are the same, it is evident that some *anterior* causes must be sought for, in order to account for this striking diversity of their pathological diathesis.

Now the 23d has long been stationed at Courbevoie, and therefore exhibits in its pathological manifestations the influences of that place alone. The 69th, on the contrary, has been only seven months at Courbevoie, but for the two previous years was in garrison at Strasbourg; and this latter regiment has evidently brought with it, and is now clearly showing, the influences of that marshy district.

This latter regiment, which was embodied at Strasbourg in 1840, yielded in the first year 73 cases of typhoid fever. After a year's residence in the marshy atmosphere of that place, the number of cases of typhoid fever sank (in 1842) to 32, less than one-half, whilst the number of intermittent fevers increased considerably. After the end of the second year, and in spite of the departure of the regiment from Courbevoie, the intermittent cases increased, so that during the first *three* months of 1843 they amounted nearly to 200, whilst, on the other hand, the number of typhoid cases fell to 2 in *seven* months.

These observations, continues M. Boudin, are not without ample precedent; for he used constantly to observe that when regiments arrived at Marseilles from the marshy localities of Corsica, the Morea, and Algiers, they remained untouched by typhoid fever, although it decimated the resident garrison of the place.

The long existing influence of a marshy soil is shown by the fact that it was by no means rare to find men affected three months after their removal from it to another.

But other diseases also, besides those arising from marsh miasmata, may exhibit themselves long after the patient has been removed from the district. Thus, regiments which proceed to Algiers from Marseilles, yield many cases of typhoid fever for months after their arrival, although that disease would be sought in vain amongst any other of the civil or military residents of the place.—*Ibid.*

